# Muscle Biopsy and Electromyography Correlation

**DOI:** 10.3389/fneur.2018.00839

**Published:** 2018-10-09

**Authors:** Elie Naddaf, Margherita Milone, Michelle L. Mauermann, Jayawant Mandrekar, William J. Litchy

**Affiliations:** ^1^Department of Neurology, Mayo Clinic, Rochester, MN, United States; ^2^Department of Internal Medicine, Mayo Clinic, Rochester, MN, United States

**Keywords:** electrodiagnostic testing, electromyography, fibrillation potentials, muscle biopsy, muscle histopathology, motor unit potentials

## Abstract

**Introduction:** In myopathies, the correlation of individual electromyographic and histopathologic findings remains poorly explored, as most previous studies have focused on the ability of muscle biopsy and electromyography to distinguish the neuropathic vs. myopathic nature of the underlying neuromuscular disease.

**Methods:** We identified 100 patients who had a muscle biopsy and electromyography performed on identical muscles. We used a detailed grading system ranging from 0- normal to 4- severe; and graded 16 histopathologic findings in each biopsy. Electromyography findings were also graded from 0 to 4 according to the standard protocol in our EMG laboratory. We used Kendall's tau for non-parametric ordinal correlation analysis.

**Results:** Fibrillation potentials correlated with atrophic, necrotic, and regenerating fibers, fibers harboring vacuoles, fiber splitting, fibers reacting for non-specific esterase, fibers with congophilic inclusions, inflammation (endoymysial and perimysial), and increased endomysial connective tissue. Short-duration motor unit potentials correlated with atrophic, necrotic, and regenerating fibers, increased endomysial connective tissue, and perimysial inflammation. Long-duration motor unit potentials correlated with fiber-type grouping. Increased phases of motor unit potentials correlated with atrophic fibers, increased endomysial connective tissue, and fibers reacting for non-specific esterase; while increased turns correlated with atrophic and regenerating fibers, increased endomysial connective tissue and target formations. Rapid recruitment correlated with regenerating fibers, perimysial inflammation, and increased endomysial connective tissue.

**Discussion:** By demonstrating a clear correlation of various electromyographic and histopathologic findings, this study improves interpreting electrodiagnostic testing in myopathies, and serves as the basis to further assess the correlation between clinical, electromyographic, and histopathologic findings.

## Introduction

Electrodiagnostic testing is a cardinal component of the evaluation of patients with suspected myopathy (1). It can help identify an ongoing myopathy, differentiating a neuropathy from a myopathy, recognizing a pattern of abnormalities that can point to a specific myopathy, determining disease activity, selecting the appropriate muscle for biopsy, and assessing response to or side effects of a pharmacological treatment. Despite the improved availability of genetic testing in the modern era, muscle biopsy and electrodiagnostic testing continue to play a key role in the diagnosis of both acquired and inherited neuromuscular disorders. However, associating specific electromyographic (EMG) findings with histopathologic correlates remains challenging. One essential EMG element clinicians rely on in their diagnostic approach is presence or absence of abnormal spontaneous discharges such as fibrillation potentials, myotonic discharges, or complex repetitive discharges (2). These findings can be associated with a wide spectrum of neuromuscular disorders encompassing neuropathic and myopathic disorders. For instance, fibrillation potentials are thought to be caused by membrane potential instability in denervated skeletal muscle (3). Similarly in myopathies, separating a segment of the muscle fiber from its endplate, as can be seen in fiber splitting or focal necrosis, can result in fibrillation potentials (4). Nonetheless, in different electrodiagnostic laboratories, the presence of fibrillation potentials may be interpreted in different ways and as a correlate of: membrane instability, active myopathy, inflammation or denervation.

Previous studies have focused on the ability of muscle biopsy and EMG to distinguish the neuropathic vs. myopathic nature of an underlying neuromuscular disease, (5–9) with rare attempts to correlate individual EMG abnormalities with specific muscle histopathology (10–12). However, this type of correlation would deepen our knowledge of disease mechanisms and how they translate into various laboratory tests. From clinical standpoint, it would allow physicians to better interpret EMG findings, understand the correlation of EMG and pathological abnormalities, and subsequently make more informed decisions on diagnosis and therapy.

## Methods

With Mayo Clinic Institutional Review Board approval, we reviewed our database for patients who underwent a muscle biopsy between October 2015 and October 2016 to identify 75 eligible patients. For a subject to be included, EMG and muscle biopsy had to be performed on the same muscle on opposite sides if the patient had symmetric weakness (for instance, muscle biopsy of the right biceps, and needle EMG of the left biceps), or on the same side if EMG was performed 3 to 6 months prior to the biopsy after verifying clinical stability of the muscle strength in that timeframe (for instance, right biceps needle examination performed 3 months prior to a right biceps biopsy). As certain rare disorders were expected to be under-represented compared to more common myopathies such as sporadic inclusion body myositis (sIBM), we also identified 25 patients with rare disorders (mitochondrial myopathy, lipid and glycogen storage diseases, myofibrillar myopathy, and vacuolar myopathy) fulfilling the same inclusion criteria, but evaluated prior to October 2015. We included under the histopathologic diagnosis of “vacuolar myopathy” the muscle specimens where the predominant feature consisted of abundant vacuoles, whether rimmed (myopathy with rimmed vacuoles) or not. To avoid the controversial term of polymyositis, the term “inflammatory myopathy with autoaggressive features” was used to describe muscle specimens with inflammatory cells invading non-necrotic fibers and lacking other histopathologic features of inclusion body myositis.

Per our Muscle laboratory protocol, muscle biopsy specimens were frozen in isopentane-cooled in liquid nitrogen with serial 10 μm sections stained with hematoxylin-eosin, trichrome, nicotinamide adenine dinucleotide dehydrogenase (NADH), succinate dehydrogenase (SDH), cytochrome c oxidase (CCO), ATPase (at pH 4.3, 4.6, and 9.4), acid phosphatase, myophosphorylase, periodic acid-Schiff (PAS), oil-red O, non-specific esterase (NSE), and Congo red. Muscle biopsy slides were then reviewed by light microscopy and Congo red stained sections were viewed under rhodamine optics to better visualize amyloid deposits. Seventeen clinically relevant histopathologic findings were graded on each specimen. These included atrophic fibers defined as fibers having a diameter less than 25 μm; necrotic fibers; regenerating fibers; fiber splitting; fibers harboring vacuoles; ragged-red fibers; CCO negative fibers; fibers with target formations; fibers with increased glycogen content; fibers reacting for NSE; fibers with congophilic inclusions; amount of endomysial connective tissue; perimysial inflammation; endomysial inflammation; fiber type grouping, type 1 and type 2 fiber atrophy. With the lack of any published validated scale for muscle biopsy grading and to avoid utilizing a binary present/absent system, we, the authors, established a grading scale based on our clinical expertise and in keeping with the practice of our Muscle laboratory. The grading scale ranged from 0 (normal) to 4 (severe) as detailed in Table 1. As fiber atrophy related to denervation is better reflected by the presence of fibers reacting for NSE, we counted all fibers with a diameter less than 25 μm as atrophic fibers regardless of their shape (rounded vs. triangular). For better accuracy, the grade was based on reviewing the full specimen rather than sampling.

**Table 1:** Grading criteria.

**Grading type**	**Histopathologic finding**	**Grade**
Quantitative	Necrotic fibers; regenerating fibers; fiber splitting; fibers harboring vacuoles; ragged-red fibers; cytochrome c oxidase negative fibers; fibers with target formations; fibers with increased glycogen content; fibers over-reacting to non-specific esterase; fibers with congophilic inclusions	0: Normal
		1 or rare: 3 or less per biopsy
		2 or mild: more than 3 per biopsy, less than 1 per 10X-power field
		3 or moderate: 1 or more per 10X-power field, less than 2 per 10X-power field
		4 or severe: 2 or more per 10X-power field
	Atrophic fibers	0: Normal
		1 or rare: Occasional atrophic fibers, less than 1 per 10X power field
		2 or mild: scattered atrophic fibers occurring singly, or in pairs, about 1 fiber or pair per 10X power field
		3 or moderate: atrophic fibers forming small groups of up to 5 fibers per group, about 1 small group per 10X power field
		4 or severe: atrophic fibers forming large groups (more than 5 fibers per group), about 1 large group per 10X power field.
Semi quantitative	Inflammation:	0: no inflammation
		1 or rare: minimal scattered inflammation
		2 or mild: 1 small collection per 5X-power field
		3 or moderate: 2 small collections per 5X-power field
		4 or severe: 3 small collections per 5X-power field
Qualitative	Type 1 fiber atrophy	0: Normal
		2: mild (type 1 fibers have a mildly smaller diameter than type 2 fibers)
		4: severe (type 1 fibers have a markedly smaller diameter than type 2 fibers)
	Type 2 fiber atrophy	0: Normal
		2: mild (type 2 fibers have a mildly smaller diameter than type 1 fibers)
		4: severe (type 2 fibers have a markedly smaller diameter than type 1 fibers)
	Endomysial connective tissue	0: normal
		1: mildly increased focally
		2: mildly increased
		3: moderately increased
		4: severely increased
	Fiber type grouping	0: random distribution of histochemical fiber types
		1 or rare: one small group
		2 or mild: occasional grouping
		3 or moderate: frequent grouping
		4 or severe: most fascicles display fiber type grouping

Per our electrodiagnostic laboratory routine, nerve conduction studies were obtained first, with at least one motor and one sensory nerve conduction study from one upper and one lower limb. EMG was then performed using a concentric needle and limiting the examination to one side allowing biopsy of contralateral side. A minimum of 20 motor unit potentials, recorded at low levels of activation were evaluated using a semi-quantitative approach and graded on a scale from 0 (normal) to 4 (severe) using the Mayo grading system (2). Each study included at least a proximal and a distal muscle from one upper and one lower limb in addition to the thoracic paraspinals. Muscle selection is based on clinical examination to include clinically-involved muscles. As nerve conduction studies are most often obtained recording from hand and foot muscles, which are rarely amenable to biopsy, and as the goal of the study is to correlate muscle biopsy and EMG findings in an individual muscle, the results of the nerve conduction studies were not included in our analysis. We reviewed the EMG findings for each patient. Fibrillation potentials, short duration motor unit potentials, long duration motor unit potentials, increased phases and turns, and rapid recruitment were graded. In our laboratory, positive sharp waves are counted as fibrillation potentials. Myotonic discharges were graded as 0 if absent and 1 if present. As there is no standard grading and a wide range of variability of the motor unit potential amplitude, amplitude analysis was not included in our study. At the time of muscle biopsy data evaluation, the reviewer (EN) was blinded to the EMG results.

### Statistical analysis

Non-parametric ordinal correlation analysis was performed using Kendall's tau (Kendall's rank correlation coefficient) which is a non-parametric measure of the degree of concordance between two variables measured on an ordinal scale.

## Results

### Diagnoses

**Figure 1:**
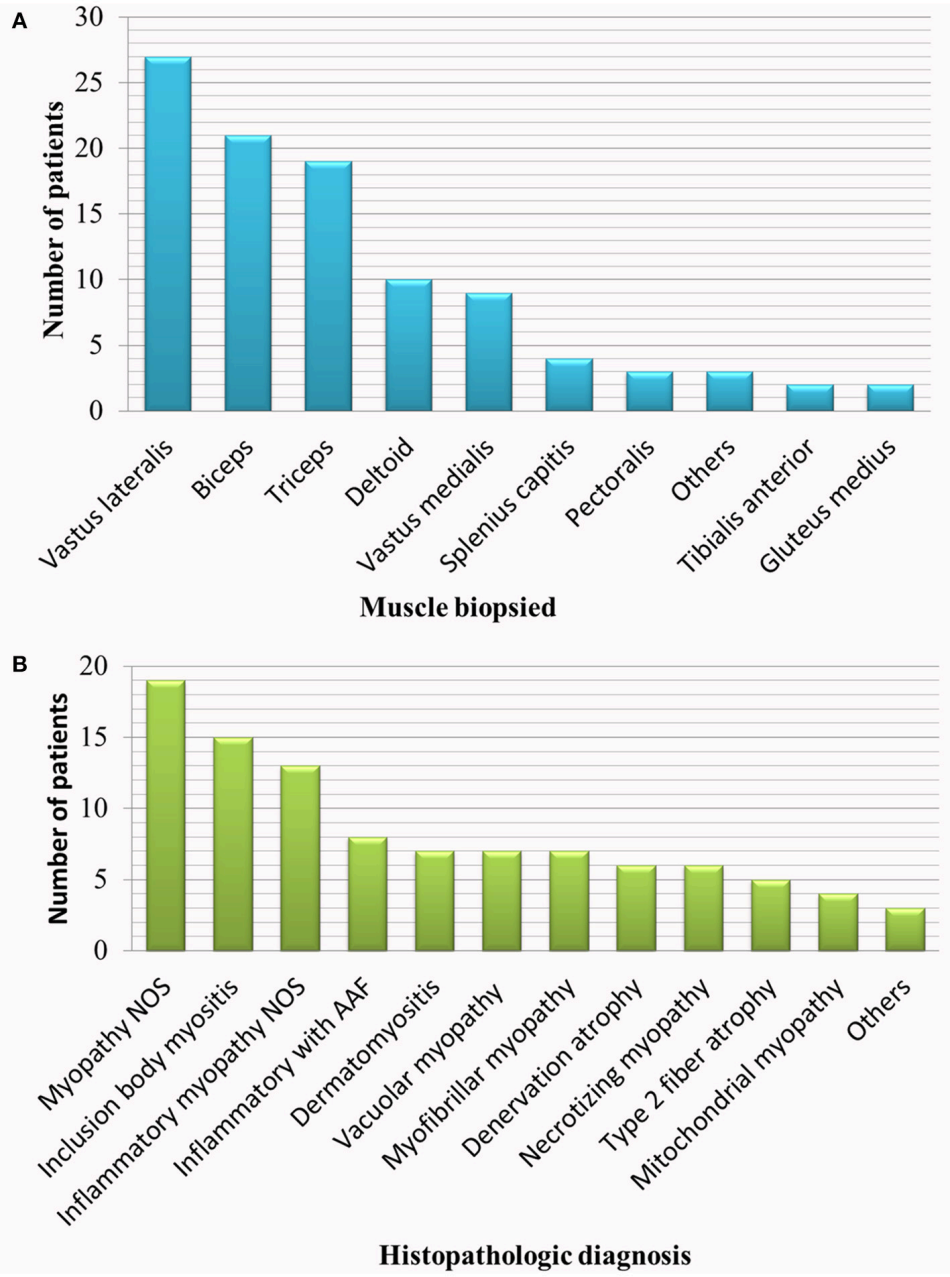
Studied population. **(A)** Muscle biopsied, **(B)** histopathologic diagnosis. AAF, auto-aggressive features; NOS, not otherwise specified.

We identified 100 patients fulfilling the inclusion criteria. Ninety six patients had biopsy and EMG performed on the same muscle on the opposite side, and four patients on the same muscle and on the same side. EMG preceded the biopsy by a mean of 22 days (95% confidence interval: 15.6–29 days). In eighteen patients, EMG and muscle biopsy were performed while the patient was on an immunomodulatory drug. The biopsy specimens had diverse histopathologic diagnoses as shown in Figure 1. Although it is not uncommon to have more than one histopathologic diagnosis revealed by a single muscle biopsy, only the predominant finding is listed in the figure. One patient with the histopathologic diagnosis of inflammatory myopathy not otherwise specified (NOS) and one patient with inflammatory myopathy with auto-aggressive features were adjudged, on clinical grounds, to have probable dermatomyositis (typical rash and interstitial lung disease) and sIBM (clinically-determined IBM with elevated cytosolic 5′-nucleotidase 1A antibodies) respectively. One patient with myopathy NOS was genetically diagnosed with *RYR1* myopathy. Among patients with vacuolar myopathy, one had Pompe disease and five had myopathy with rimmed vacuoles (hereditary inclusion body myopathy): two of these patients had pathogenic mutations in *VCP*, one in *GNE* and one in *TIA1*. The six patients with necrotizing myopathy and one patient with myopathy NOS were diagnosed with necrotizing autoimmune myopathy based on clinical history, response to pharmacological treatment and 3-hydroxy-3-methylglutaryl–coenzyme A reductase or signal recognition particle antibody positivity.

### Electromyographic findings

**Figure 2:**
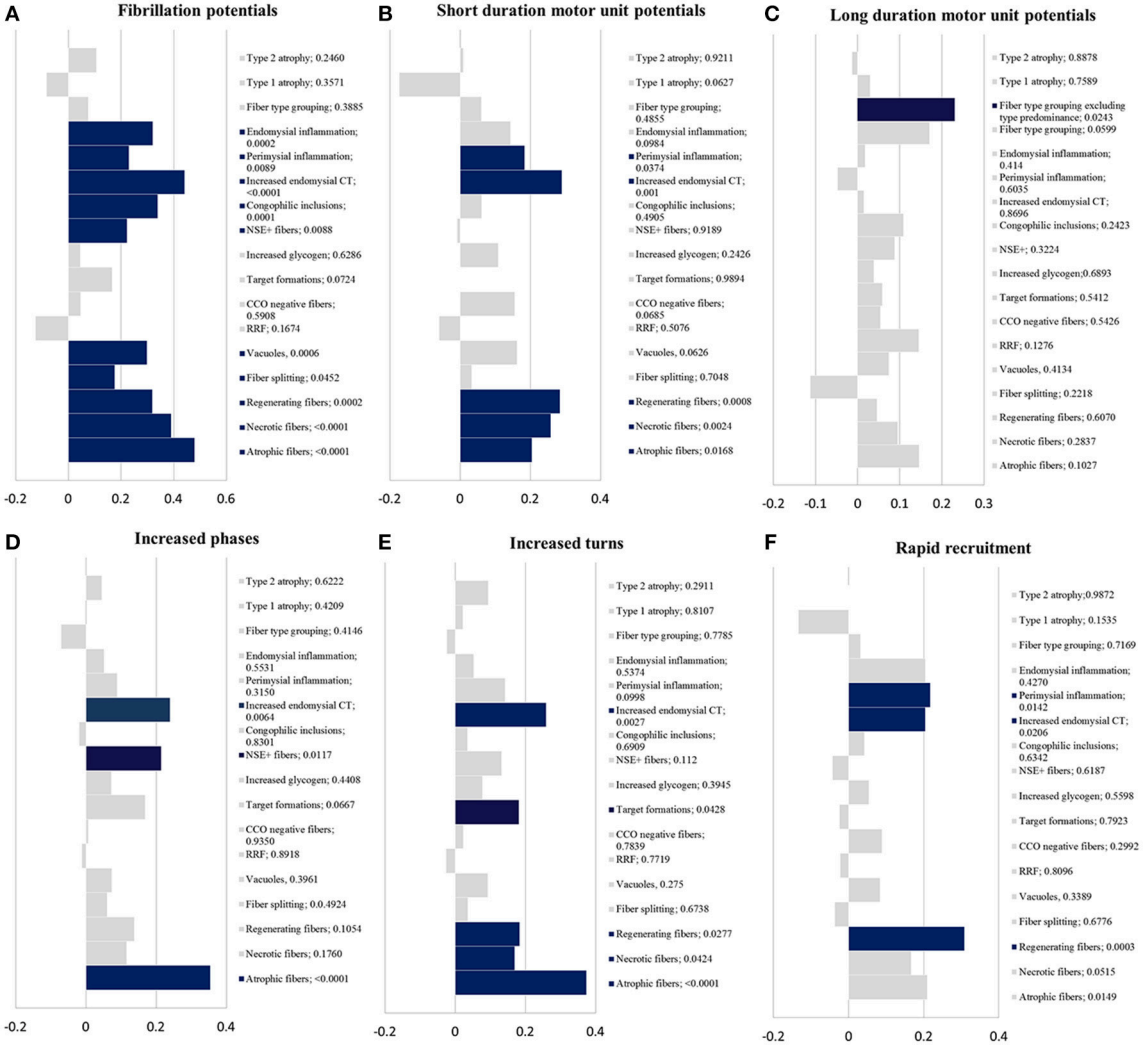
Statistical results. Kendall-tau *t*-value (*x*-axis) vs. histopathologic findings with correspondent *p*-value (*y*-axis); blue bars have a *p* < 0.05 and gray bars ≥ 0.05. **(A)** fibrillation potentials; **(B)** short duration motor unit potentials; **(C)** long duration motor unit potentials; **(D)** increased phases; **(E)** increased turns; **(F)** rapid recruitment. CCO, cytochrome c oxidase; CT, connective tissue; NSE+ fibers, fibers reacting for non-specific esterase; RRF, ragged-red fibers.

1. Fibrillation potentials
2. Sixty eight patients had fibrillation potentials on EMG. The percentage of patients with fibrillation potentials in each histopathologic diagnosis were as follows: 100% in sIBM, dermatomyositis and myofibrillar myopathy; 77% in inflammatory myopathy NOS, 67% in denervation with or without reinnervation, 63% in inflammatory myopathy with auto-aggressive features, 57% in vacuolar myopathy, 42% in myopathy NOS, 25% in mitochondrial myopathy and 16% in type 2 fiber atrophy.
3. There was a correlation between the presence of fibrillation potentials on EMG and the following histopathologic findings (Figure 2A): atrophic fibers, necrotic fibers, regenerating fibers, fiber splitting, fibers harboring vacuoles, fibers reacting for NSE, fibers with congophilic inclusions, increased endomysial connective tissue and inflammation (endomysial and perimysial).
4. Short duration motor unit potentials
5. Eighty five patients had evidence of short duration motor unit potentials on EMG. There was a correlation between short duration motor unit potentials and the following histopathologic findings (Figure 2B): atrophic fibers, necrotic fibers, regenerating fibers, increased endomysial connective tissue, and perimysial (not endomysial) inflammation.
6. Long duration motor unit potentials
7. Nineteen patients had long duration motor potentials. Most commonly, long duration motor unit potentials were seen in patients with histopathologic features of an inclusion body myopathy (6 patients with sIBM and one patient with VCP myopathy). Thirty three patients had fiber-type grouping and 19 additional patients had fiber-type predominance. There was a significant correlation between long duration motor unit potentials and fiber type grouping (*p* = 0.0243) but not with fiber type predominance (*p* = 0.4566) (Figure 2C).
8. Increased phases and turns of the motor unit potentials
9. There was a correlation between increased phases and the presence of (Figure 2D): atrophic fibers, fibers reacting for NSE and increased endomysial connective tissue. Regarding increased turns, there was a correlation with the presence of atrophic fibers, regenerating fibers, target formations, and increased endomysial connective tissue (Figure 2E).
10. Rapid recruitment
11. There was a correlation between rapid recruitment and the presence of (Figure 2F): regenerating fibers, perimysial inflammation, and increased endomysial connective tissue.
12. Myotonic discharges
13. Myotonic discharges were recorded in only nine patients: necrotizing myopathy (4 patients), sIBM (1), myopathy with rimmed vacuoles (1), Pompe disease (1), MFM (1) and inflammatory myopathy NOS (1). They did not correlate with any of the studied histopathologic findings. A statistically non-significant correlation (trend) was noted between myotonic discharges and necrotic fibers (*p* = 0.0637), cytochrome C oxidase negative fibers (*p* = 0.0542) and glycogen accumulation (*p* = 0.0794).

**Figure 3:**
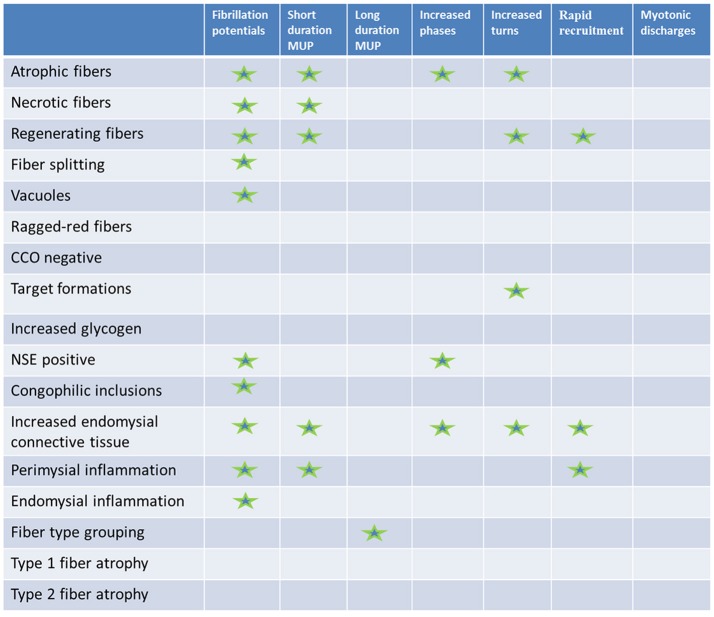
Results summary. Histopathologic (rows) vs. electromyographic (columns) findings: statistically significant correlations are marked with a star.

The results are summarized in Figure 3.

## Discussion

This is the first study to demonstrate in a systematic semi-quantitative manner the correlation between various EMG and histopathologic findings. A similar methodology could be applied to individual disorders (i.e., dermatomyositis) allowing to draw more detailed conclusions, that may help in diagnosis and treatment decisions. Adopting a detailed pathological grading system and subsequent ordinal correlation statistical analysis were crucial. As in practice, the detection of a specific histopathologic finding occurring rarely is often of unknown clinical significance and does not imply a specific pathological diagnosis (for instance, a single fiber with nemaline rods does not imply the diagnosis of a nemaline myopathy). Furthermore, due to random sampling, the more frequent an abnormality is, the more likely it will be detected by electrodiagnostic testing and on muscle biopsy. Hence, a binary absent-or-present grading system would not be sufficient. In our practice, we select a muscle to biopsy based on clinical involvement, electrodiagnostic testing and sometimes muscle imaging. This resulted in a wide variety of muscles biopsied and the diverse clinical and histopathologic diagnoses enriched the studied sample.

Fibrillation potentials correlated with a wide spectrum of histopathologic findings including: inflammation, active myopathy (necrosis and regeneration), fiber disruption (fiber splitting, vacuoles and congophilic inclusions), chronicity (increased endomysial connective tissue) and denervation (fibers reacting for NSE). While the presence of atrophic fibers highly correlated with fibrillation potentials on EMG, type 1, or type 2 fiber atrophy in isolation did not.

Therefore, the presence of fibrillation potentials should be interpreted with caution and should not be considered as a surrogate for just inflammation or disease activity. The percentage of patients with fibrillation potentials per histopathologic diagnosis in this study may not necessarily reflect the true prevalence of fibrillation potentials in such disorder, in part due to the fact that a clinically-involved muscle with fibrillation potentials on EMG is usually a preferred target for biopsy.

The duration of the motor unit potentials is a key component to determine the nature of an underlying process. However, it is not uncommon to have mixed short and long duration motor unit potentials especially in sIBM (13). In our study, short duration motor unit potentials were associated with the loss of electrically excitable muscle fibers from the motor unit (14). On the other hand, long duration motor unit potentials solely correlated with histochemical fiber type grouping but not with fiber type predominance. In a normal muscle, there is random distribution of histochemical fiber types. In reinnervated skeletal muscles, the checkerboard appearance is disrupted and replaced by grouping of fibers of the same histochemical type, as fibers innervated by a single motor unit become of the same histochemical type (12). Sometimes, the presence of long duration motor unit potentials may be interpreted as a sign of chronicity of the myopathy, which did not hold true in our study. Similarly to previous reports, (11) increased phases and turns were associated with histologic signs of denervation (fibers reacting for NSE, target formations and atrophic fibers). It remains unclear why short duration motor unit potentials and rapid recruitment correlated with perimysial and not endomysial inflammation despite both equally represented in our cohort (32% of samples had evidence of perimysial and 34% of endomysial inflammation).

Limitations of the present study include the retrospective design and the mostly semi-quantitative nature of the data. Furthermore, the pathological findings are not completely independent, as they tend to occur in patterns such as the association of necrotic and regenerating fibers. To better interpret histopathologic and EMG findings, the clinical context should be considered. For the purpose of this study, we limited the clinical information to the clinical diagnosis, the use of immunomodulatory drugs, time lag between biopsy and EMG, and ensuring strength stability between the two procedures.

In this study, we demonstrated the correlation between various EMG and histopathologic findings. The results reflect the reasonable accuracy of the used methods especially the histopathologic grading system. Therefore, the findings and the methodology could serve as a platform for future studies to further assess the correlation with clinical or radiographic data as well as to better understand the muscle biopsy-EMG correlation in individual disorders.

## Author contributions

EN, MaM, MiM, and WL: study concept and design; EN: acquisition of data; EN, MaM, MiM, JM, and WL: data analysis and interpretation; EN and JM statistical plan and analysis. EN drafting the manuscript. EN, MaM, MiM, JM, and WL critical revision of the manuscript for important intellectual content.

### Conflict of interest statement

The authors declare that the research was conducted in the absence of any commercial or financial relationships that could be construed as a potential conflict of interest.
